# Short-term follow-up of exercise training program and beta-blocker treatment on quality of life in dogs with naturally acquired chronic mitral valve disease

**DOI:** 10.1590/1414-431X20154568

**Published:** 2015-08-04

**Authors:** M. Marcondes-Santos, A.P. Mansur, F.S. Fragata, C.M.C. Strunz

**Affiliations:** 1 Hospital Veterinário Sena Madureira, São Paulo, SP, Brasil; 2 Instituto do Coração, Hospital das Clínicas, Faculdade de Medicina, Universidade de São Paulo, São Paulo, SP, Brasil

**Keywords:** Chronic mitral valve disease, Beta-blocker, Exercise training, Dogs

## Abstract

This study aimed to evaluate the effects of carvedilol treatment and a regimen of supervised aerobic exercise training on quality of life and other clinical, echocardiographic, and biochemical variables in a group of client-owned dogs with chronic mitral valve disease (CMVD). Ten healthy dogs (control) and 36 CMVD dogs were studied, with the latter group divided into 3 subgroups. In addition to conventional treatment (benazepril, 0.3-0.5 mg/kg once a day, and digoxin, 0.0055 mg/kg twice daily), 13 dogs received exercise training (subgroup I; 10.3±2.1 years), 10 dogs received carvedilol (0.3 mg/kg twice daily) and exercise training (subgroup II; 10.8±1.7 years), and 13 dogs received only carvedilol (subgroup III; 10.9±2.1 years). All drugs were administered orally. Clinical, laboratory, and Doppler echocardiographic variables were evaluated at baseline and after 3 and 6 months. Exercise training was conducted from months 3-6. The mean speed rate during training increased for both subgroups I and II (ANOVA, P>0.001), indicating improvement in physical conditioning at the end of the exercise period. Quality of life and functional class was improved for all subgroups at the end of the study. The N-terminal pro-brain natriuretic peptide (NT-proBNP) level increased in subgroup I from baseline to 3 months, but remained stable after training introduction (from 3 to 6 months). For subgroups II and III, NT-proBNP levels remained stable during the entire study. No difference was observed for the other variables between the three evaluation periods. The combination of carvedilol or exercise training with conventional treatment in CMVD dogs led to improvements in quality of life and functional class. Therefore, light walking in CMVD dogs must be encouraged.

## Introduction

Congestive heart failure is characterized by activation of the neurohumoral axis, with increased release of norepinephrine (NE), angiotensin II, and arginine vasopressin (1). Dogs with naturally acquired mitral valve disease exhibit a positive correlation between increased sympathetic activity and severity of heart failure (2).

The deleterious effects of sympathetic nervous system hyperactivity may be prevented by the use of beta-blockers in dogs with heart failure as these drugs exert protective effects on cardiomyocytes and are effective in reducing ventricular dysfunction (3,4). Metoprolol administration in dogs with experimentally induced mitral regurgitation promotes a decrease in cardiac interstitial NE (5). An effective dose of carvedilol has been established using healthy dogs (6 7 8 9), and some authors have reported the possible beneficial effects of beta-blocker treatment in dogs with chronic mitral valve disease (CMVD) (10,11).

Our preliminary paper demonstrated improvements in quality of life and functional class and a decrease in blood pressure (BP) in CMVD dogs after 3 months of carvedilol treatment (12). Recent studies of human heart failure have shown that an exercise protocol including regular aerobic physical training may be helpful for sympathetic tonus modulation. According to a previous meta-analysis, hospital admission and mortality rates are significantly reduced in chronic heart failure patients who participate in physical exercise, which may promote a reduction in sympathetic tonus and an increase in vagal tonus after training (13). Other authors have demonstrated the benefits of exercise training after human mitral valve replacement through improvements in quality of life and exercise tolerance (14,15). No similar studies have yet been conducted in dogs with cardiac failure, although previous studies have shown that it is safe to use an electric treadmill for physical conditioning in dogs (16,17).

The objective of the present study was to evaluate the effects of carvedilol and a regimen of supervised aerobic exercise training on quality of life and other clinical, echocardiographic, and biochemical variables in a group of client-owned dogs with CMVD.

## Material and Methods

### Animals

A prospective controlled study was conducted using 36 client-owned dogs that were diagnosed with CMVD at the Cardiology Service of the Hospital Veterinário Sena Madureira (São Paulo, SP, Brazil). In addition, 10 healthy dogs (5 females and 5 males: 2 beagles, 1 cocker spaniel, 1 dachshund, 1 Lhasa Apso, 1 pit bull, 1 poodle, 1 schnauzer, 1 pug and 1 mixed-breed dog; mean age 7.2±2.6 years) were selected for comparisons of the study variables.

Dogs with mitral regurgitation and left atrial enlargement were chosen and classified as grade I through IV according to the New York Heart Association Functional Classification scoring system that had been modified for veterinary use (18); grading was based on the historical severity of the heart failure signs and the physical, radiographic, and echocardiographic findings. Briefly, functional class I was defined as heart murmur of mitral origin with no signs of heart enlargement and no limitation to physical activity; class II: slight limitation to physical activity with varying degrees of heart enlargement without clinical signs; class III: marked limitation of physical activity with radiologic signs of congestive heart failure; class IV: severe limitation of physical activity with radiologic signs of congestive heart failure (18).

After selection, the groups of dogs were balanced for gender and functional classification in an attempt to avoid the influence of different clinical conditions between the groups on the results.

**Figure 1:**
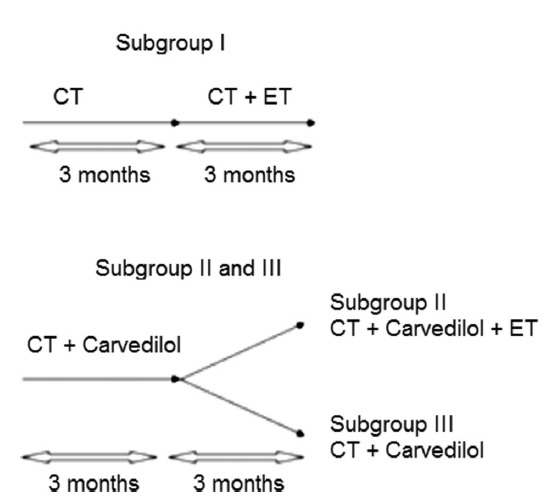
Study design. CT: conventional treatment; ET: exercise training.

Seven dogs were assigned to functional class I, 14 were assigned to class II, 12 were assigned to class III, and 3 were assigned to class IV. The dogs were divided into the following three subgroups according to the treatment protocol used (Figure 1): subgroup I included 13 dogs (4 females and 9 males; 1 basset hound, 1 pinscher, 10 poodles, and 1 mixed-breed dog) with a mean age of 10.3±2.1 years; subgroup II included 10 dogs (4 females and 6 males; 1 beagle, 1 pinscher, 5 poodles, and 3 mixed-breed dogs) with a mean age of 10.8±1.7 years; subgroup III included 13 dogs (5 females and 8 males; 1 cocker spaniel, 1 dachshund, 1 Lhasa Apso, 1 pinscher, 8 poodles, and 1 mixed-breed dog) with a mean age of 10.9±2.1 years.

The Ethics Committee of the Instituto do Coração, Faculdade de Medicina, Universidade de São Paulo, approved this study, which was performed in compliance with the guidelines for reporting of *in vivo*experiments in animal research (19). Written informed consent was obtained from each client, who was informed about the drugs and exercise training protocols used and their possible side effects. The differences between the protocol treatments were omitted, so the clients were blind to the identity of the drug being studied and to the protocols used to treat the other study dogs.

### Study design

The study design is shown in Figure 1. The clinical evaluation comprised physical examination, electrocardiography (ECG), thoracic radiography, plasma and serum biochemical analysis, and two-dimensional, M-mode, spectral pulsed Doppler echocardiography. All CMVD dogs underwent the above evaluation at baseline (T0) and after 3 (T1) and 6 (T2) months.

The conventional treatment used for all animals was as follows: benazepril (0.3-0.5 mg/kg once a day) for the dogs with a classification of functional class I, and benazepril and digoxin (0.0055 mg/kg twice daily) for the dogs with functional class ≥II. The diuretic was furosemide (mean dose of 2.5 mg/kg twice daily) for dogs in functional classifications II and III, and in association with spironolactone (2 mg/kg twice daily) and with hydrochlorothiazide (2 mg/kg twice daily) for dogs in functional class IV (1 dog in each subgroup). All drugs were administered orally.

The dogs in subgroup I received the conventional treatment for the entire duration of the study. Those in subgroups II and III received the conventional treatment with the addition of carvedilol (0.3 mg/kg orally twice daily) during the 6-month study period. The initial dose of carvedilol was 0.15 to 0.2 mg/kg orally twice daily for 1 week; after evaluating BP and heart rate (HR), the dose was raised to 0.3 mg/kg orally twice daily.

At 3 months after initiation of the study (T1), subgroups I and II began a regimen of supervised aerobic training on a treadmill twice weekly at the veterinary heart rehabilitation room.

The functional evaluation of cardiac health (FETCH) questionnaire score used to evaluate quality of life was established based on information obtained from the client at T0, T1, and T2. As previously described, the questionnaire was developed based on widely accepted clinical signs of cardiac disease in dogs (20); it comprised 17 questions answered by the client, who graded symptom severity on a scale ranging from 0 to 5 (for which 0=few and 5=many symptoms). A higher score indicated a poorer health-related quality of life. The questions were mainly related to respiratory symptoms, mobility difficulties (such as walking and climbing stairs), physical activity, irritability, appetite, sleepiness, and reactions such as frequent urination and vomiting. The correlation between the FETCH score and the functional class was assessed for the 36 dogs at T0 using the Spearman rank test (r=0.729; P<0.0001).

NE was assessed using high-performance liquid chromatography with an electrochemical detector (11) (Waters Corp., USA), and sodium was measured using a selective electrode (Dimension RxL, Siemens Health Care Diagnostics, USA). Specific kits for automated equipment (Dimension RxL) were used to determine the urea, creatinine and troponin I levels (Immulite 1000, Siemens Health Care Diagnostics, Germany). Plasma N-terminal pro-brain natriuretic peptide (NT-proBNP) was measured using a commercial enzyme-linked immunosorbent assay kit that was specific for canine NT-proBNP (Vet Sign Canine CardioSCREEN NT-Pro-BNP Kit, Guildhay, UK).

### Echocardiographic and electrocardiographic evaluation

The echocardiographic examination was performed using an ultrasound system equipped with a 5-MHz microconvex transducer (Aloka SSD 650 ultrasound system, Aloka Inc., Japan) (21). The left ventricular dimensions and left atrium (LA) were indexed to the aortic root diameter (Ao). BP was measured indirectly three times by vascular Doppler examination (Medmega DV-610, Medmega, Brazil) with the dogs positioned in lateral recumbency.

The M-mode echocardiographic variables studied included diastolic interventricular septal thickness, diastolic left ventricular wall thickness, diastolic left ventricular internal dimension (LVIDd) to Ao ratio, systolic left ventricular internal dimension (LVIDs) to Ao ratio, ejection fraction, fractional shortening (FS), and LA/Ao. The FS values were calculated based on the equation FS=([LVIDd-LVIDs]/LVIDd)×100 (21).

The severity of mitral regurgitation was estimated using spectral pulsed Doppler ultrasonography based on the percentage of the LA occupied by the regurgitant jet (mild, <20%; moderate, 20 to 50%; or severe, >50%) (22). HR was evaluated using an ECG machine (model E.C.G.-6, Ecafix, Brazil).

### Aerobic exercise training protocol

To conduct this protocol, a veterinary heart rehabilitation room was designed with environmental temperature control, oxygen therapy equipment, emergency drugs, and a defibrillator. A suitable dog treadmill with digital speed control was used (Fisicow Dog, Brazil). Cardiac monitoring was performed using ergometric equipment (Cardiobyt, Brazil) connected to a microcomputer and an HR monitor (Polar Electro, USA). To improve attendance by clients at the exercise training sessions, 20-min exercise sessions were conducted twice weekly for 3 months.

### Supervised aerobic training protocol

The animals in subgroups I and II were adapted to walking on the treadmill prior to the exercise sessions. The clients sat in front of their dogs during this adaptive exercise period (10 min at a minimum speed of 2.1 km/h), with or without offering canine treats to motivate them to perform the exercise.

After treadmill adaptation, an effort test was performed. The highest heart rate (HHR) recorded during the effort test was the value obtained when the animal showed signs of tiredness, including attempting to stop exercising and experiencing increased respiratory frequency or breathlessness similar to what had been previously described (17,23). When the animal showed signs of restlessness and an HR that was higher than the basal value, the treadmill speed was decreased, and the HHR experienced during the test was recorded. If none of these signs were present, the speed was maintained or slightly raised to the point that such signs became evident. The tongue mucosa color was constantly assessed, and if any color alteration was noticed (cyanosis or paleness), the speed was gradually decreased until normal color returned, even in the absence of signs of restlessness or breathlessness. The effort test was performed by gradually increasing the treadmill speed by approximately 1 km/h every 2 min above the 2.1 km/h starting speed.

The training heart rate (THR) was calculated for aerobic exercise using the reserve HR. The reserve HR was calculated as the difference between the HHR and the heart rate at rest (RESTHR) during the effort test. The THR was calculated as 50% to 70% of the reserve HR in addition to the RESTHR according to the following formula: THR=(HHR-RESTHR)×50-70%+RESTHR (24).

### Ergometric test and lactate measurements

After the THR calculation was performed at T1, the dogs underwent a new ergometric test and lactate measurement to confirm the aerobic nature of the exercise practice. Blood was collected by saphenous vein puncture prior to initiation of the test (at rest) and soon after its completion, and the lactate concentration was measured using a portable device (Accusport Lactate Roche, Switzerland) (16).

Evaluations were performed at T1, 45 days afterwards, and at T2 to assess the mean speed rate (MSR) and the animal’s performance during the testing period. The aerobic training speed was considered to be the value achieved when the animal reached the THR calculated during the effort test without any significant increase in blood lactate levels after training (17). The supervised physical exercise program was initiated with 20-min training sessions performed twice weekly at the MSR calculated at T1 until the second performance test and lactate assessment at 45 days. During the second test, the MSR was again calculated, and the animals remained at this speed for another 45 days until the final tests were performed at T2.

### Statistical analysis

The data are reported as means±SD. The Kolmogorov-Smirnov normality test was used to test for normal distribution of the data pertaining to the variables. Based on these results, when a normal distribution was present, the parametric Student’s *t*-test (for independent samples) or ANOVA (when the three groups were compared) was used. When normal distribution was absent, the nonparametric Mann-Whitney test (for independent samples) or nonparametric Friedman and Kruskal-Wallis tests were used. In addition, the chi-squared and Fisher’s exact tests were used to evaluate the groups in relation to their proportions. For the survivor study, the Kaplan-Meier method was chosen. A P<0.05 was considered statistically significant. Statistical analyses were performed using the Statistical Analysis System program for Windows, version 9.2 (SAS Institute Inc., 1989-1996, USA).

## Results

The characteristics of the 36 CMVD dogs were compared with those of the healthy dogs (Table 1). Compared with the control group, the experimental group was older and exhibited higher NT-proBNP, NE, troponin I, sodium, FS, ejection fraction, LVIDd/Ao, LVIDs/Ao, and LA/Ao values.

**Table:**
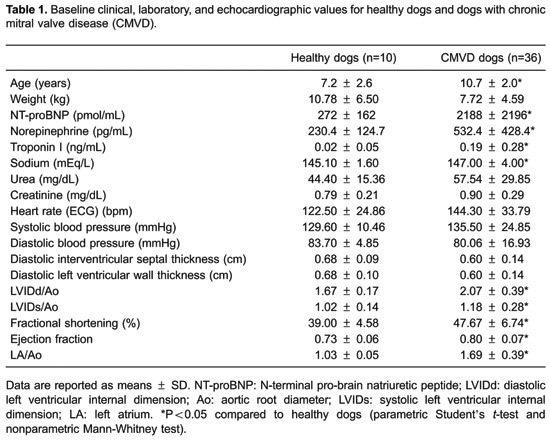

The experimental group showed differing degrees of mitral regurgitation, including mild (n=4), moderate (n=18), and severe (n=14).

The presence of adverse events during the study did not differ significantly between the subgroups, and consisted of the following: syncope and presyncope in two animals in subgroup I (15.4%), one animal in subgroup II (10%), and six animals in subgroup III (46.2%); hypotension in one animal in subgroup I (7.7%), three animals in subgroup II (30%), and six animals in subgroup III (46.2%); and absence of azotemia in subgroup I (0%) and its presence in two animals in subgroup II (20%) and three animals in subgroup III (23.1%).

Twelve animals died during the 6 months of the study, including 4 in subgroup I, 3 in subgroup II, and 5 in subgroup III. Dog survival after 6 months, as calculated using a Kaplan-Meier curve, did not differ significantly between the three subgroups (log-rank test, P=0.887).

Only those animals that survived the entire protocol period (n=9, subgroup I; n=7, subgroup II; and n=8, subgroup III) were included for statistical analysis of the variables assessed over the course of the study.

The distributions of functional classes I, II, and III among the subgroups at T0 were not significantly different (Table 2). The subgroups were also homogeneous with regard to the laboratory and ECG data (data not shown).

**Table:**
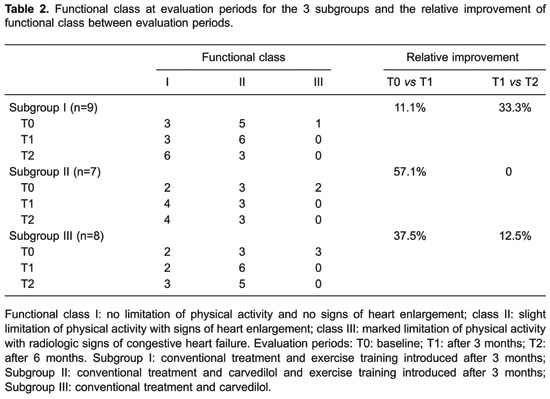

To evaluate the supervised aerobic training, the THR, MSR, and lactate values (before and soon after the test) were analyzed at three evaluation periods (T1, 45 days after starting the training, and T2).

**Figure 2:**
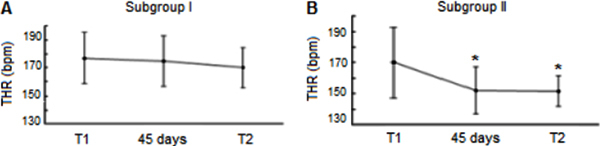
Training heart rate (THR; bpm) calculated at the three evaluated time points of the supervised physical exercise regimen. Subgroup I (*A*) showed no difference between the evaluation periods. Subgroup II (*B*) showed different progressions during the training period according to ANOVA. T1: 3 months after study initiation, T2: 6 months after study initiation. *P=0.009, THR at T1 compared with THR at 45-days for subgroup II (ANOVA); *P=0.025, THR at T1 compared with THR at T2 for subgroup II (ANOVA).

The THRs obtained for subgroups I and II differed. In subgroup I, the animals maintained a stable THR during the three evaluation periods (THR values at T1, 45 days, and T2 of 177.22±18.56, 175.00±18.20, and 170.00±14.36 bpm, respectively, P=0.091). In subgroup II (treated with a beta-blocker), the THR values observed during the evaluation periods differed significantly (T1 *vs* 45 days *vs* T2; 170.00±22.73 *vs* 152.14±15.24 *vs* 151.71±9.78 bpm, respectively, P=0.011), and the values at T1 were significantly higher than those at 45 days (P=0.009) and at T2 (P=0.025; Figure 2A and B).

**Figure 3:**
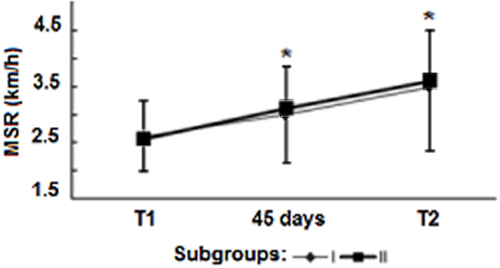
Mean speed rate (MSR) training progressions (km/h) for subgroups I and II during the study period. T1: 3 months after study initiation, T2: 6 months after study initiation. *P=0.001, T1 compared to 45-days; 45-days compared o T2; T1 compared to T2 for the two subgroups (ANOVA).

All of the animals exhibited significantly improved physical conditioning after 3 months. The training MSR increased gradually in both subgroups I and II; the values at T1, 45 days, and T2 were 2.60±0.61, 3.00±0.86, and 3.49±1.13 km/h, respectively, for subgroup I (P<0.001), and 2.56±0.68, 3.11±0.77, and 3.60±0.90 km/h, respectively, for subgroup II (P<0.001; Figure 3).

The lactate test performed before and after the ergometric tests at the different time points (T1, 45 days and T2) indicated that the training was aerobic. The values before and after the training for subgroup I were as follows: T1 before: 3.58±0.84 *vs* T1 after: 3.9±1.03 mM, P=0.398; 45 days before: 3.44±0.43 *vs* after: 3.64±0.61 mM, P=0.339; and T2 before: 3.33±0.42 *vs* after: 3.59±1.14 mM, P=0.527. The values before and after the training for subgroup II were as follows: T1 before: 2.97±0.54 *vs* after: 2.94±0.77 mM, P=0.924; 45 days before: 3.00±0.52 *vs* after: 3.30±0.65 mM, P=0.296, and T2 before: 2.66±0.21 *vs* after: 2.81±0.60 mM, P=0.376.

The FETCH score, HR, NE level, NT-proBNP level, BP and echocardiographic variables were evaluated at all of the evaluation periods, and the data for the three subgroups at each time point were compared (Table 3). There were no statistically significant differences between the three subgroups at T0.

**Table:**
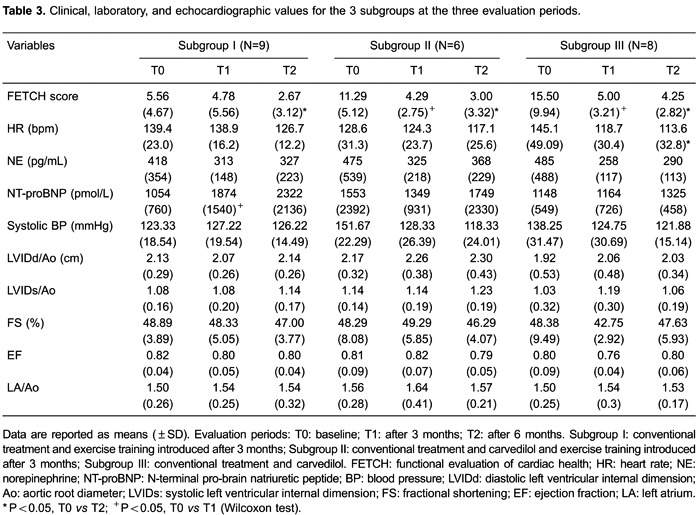

After 6 months of treatment (T2), improvement was observed in the FETCH score for all of the analyzed subgroups (T0 *vs* T2). Subgroups II and III presented better scores at T1 compared with T0.

The RESTHR was lower at T2 than at T0 for subgroup III, and there was a trend of decreasing values with increasing time for the other two subgroups. The NE levels did not show a significant difference at the three time points evaluated (T0, T1, and T2) among any of the subgroups.

The NT-proBNP level increased in subgroup I after 3 months of conventional treatment (T0 *vs* T1), but remained stable when exercise was introduced for this subgroup (T1 *vs* T2) and for the other two subgroups during the entire evaluation period.

During the study, the systolic BP did not differ among the three subgroups. In addition, stable BP values were observed for subgroup I (without carvedilol), and trends toward decreasing values were observed for subgroups II and III (with carvedilol).

Echocardiographic variables did not change during the three time points for all subgroups.

At the end of the study, the functional class of each of the three subgroups had improved to almost the same degree (approximately 50%), although a greater improvement was observed in subgroup I after the introduction of exercise. For the other two subgroups, functional class improvement occurred mainly during the first 3 months, which was associated with carvedilol treatment (Table 2).

No beneficial changes were observed when considering only the severe cases of mitral regurgitation in the three subgroups. The other clinical and laboratory variables did not differ among the treatment groups (data not shown).

## Discussion

In the present study, the use of carvedilol and a regimen of supervised aerobic training improved functional class, the FETCH score and quality of life, and stabilized the NT-proBNP level in the CMVD dogs. The NT-proBNP level increased only during the conventional treatment alone.

The characteristics of the CMVD dogs with regard to NT-proBNP, NE, troponin I, and sodium values were significantly higher than those in the healthy dogs, and were similar to those that have been reported in previous studies (2,11,25,26). It has been suggested that the sympathetic nervous system and renin-angiotensin-aldosterone activities are exacerbated in these animals. Compared with healthy dogs, the CMVD dogs showed higher LA/Ao, ejection fraction and FS values at the beginning of the study, which corroborates previous reports (11,27). It is well known that left ventricular afterload is usually reduced in compensated mitral regurgitation. Indices of systolic function such as ejection fraction and FS are inversely correlated with afterload, and consequently they tend to be normal or even slightly higher in patients with compensated mitral regurgitation (28).

Treatment of the CMVD dogs with carvedilol did not improve survival, regardless of the dosage used and whether or not its use was combined with physical exercise; this is similar to previous reports of dogs treated with the angiotensin-converting enzyme inhibitor benazepril for 6 months, and in humans with heart failure (13,29,30).

The tendency toward a decrease in systolic BP observed in the two subgroups treated with carvedilol may be associated with its vasodilatory effect, and an increase in the number and contractile force of cardiomyocytes (4,12,). However, the reduced values of systolic BP at T2 in subgroup II (carvedilol plus conventional therapy plus exercise training) were not significantly different from the results obtained for subgroup I (without carvedilol), meaning the addition of the beta-blocker to the therapy was not an obstacle to participation in an exercise training program.

Our results suggest that an individual carvedilol dose may be used to treat CMVD dogs. It is recommended that the treatment is initiated at a low dose (0.15 mg/kg twice daily), which may be gradually increased each week based on renal function, BP, and HR to an expected individual dose of up to 0.3 mg/kg administered twice daily (31).

The aerobic exercise performed by the dogs resulted in good physical conditioning at the end of the study, as shown by the increase in MSR in both of the subgroups. The THR values did not increase as expected along with the improved exercise performance; in contrast, they decreased in subgroup II, which was most likely due to the effects of the beta-blocker administered to these dogs during the entire exercise training period.

The lower resting HR in subgroup III at 6 months (T2) could have been a result of the beta-adrenergic blocking action of carvedilol, despite the non-significant decreasing trend observed in subgroup II. This trend was also detected in subgroup I just after the addition of the supervised exercise training to the conventional treatment. The small number of animals assessed could have been the reason for the non-significant results. The highly variable NE levels and small number of animals were likely responsible for the absence of significant changes in the catecholamine plasma levels observed during the study.

Improvements in quality of life and functional class and stabilization of the NT-proBNP level were similar for subgroups II and III. This may have been associated with the carvedilol treatment, as no improvement was observed in the dogs treated with the combination of the exercise training program and drug therapy in subgroup II.

However, clinical improvements were noticeable in subgroup I just after initiation of exercise training, as shown by a decrease in the FETCH score, an improvement in functional class, and stabilization of the NT-proBNP level. It is known that exercise training prevents further damage to the myocardium even though NT-proBNP levels will not change or may even decrease (34).

Other authors have reported a decrease in HR and an improvement in functional class in human patients with dilated cardiomyopathy after adding carvedilol to their treatment regimens; this is most likely due to the beta-blocker and its adrenergic, antioxidant, antiproliferative, and antiarrhythmic activities (35).

The improvement in quality of life reported here corroborates previous studies of humans in which the effects of an exercise training program were assessed after surgical treatment for mitral valve disease (14,15).

It is worth noting that although the two treatments (carvedilol and exercise training) interfere with the sympathetic tonus modulation, there was no additive effect from using both therapeutic treatments at the same time (subgroup II). However, a longer training program may change this result.

Notably, despite the similar clinical improvements experienced by all of the subgroups at the end of the study, subgroup I had a lower percentage of intercurrences, and although this difference was non-significant, it must be considered. Some authors have related the intercurrences to the inotropic and negative chronotropic actions of carvedilol, and have recommended its addition to the treatment regimen for CMVD dogs already stabilized on other heart failure medications (8,27,36). The adverse events observed in this study corroborate this suggestion.

A major problem at the beginning of the study involved the development of a safe condition of aerobic exercise training in dogs with cardiac failure considering the absence of a standard program and the low tolerance of these dogs to physical activity. Another important point was the compliance of the client-owner to training-related visits, which had to be kept to a minimum. In this study, we implemented 24 visits in 12 weeks. At the end of the study the dogs were in good physical condition, validating the efficiency of the aerobic exercise training program. An extension of the program in terms of increased frequency and length of training sessions will probably produce even better results. So light walking for CMVD dogs should be encouraged at least two to three times a week for about 20 to 30 min. An important limitation of our study was the small number of dogs used. Future studies are needed to confirm our findings.

In conclusion, the association of carvedilol or exercise training with conventional treatment in CMVD dogs led to improvements in quality of life and functional class. Therefore, light walking must be encouraged for CMVD dogs.
